# “She was finally mine”: the moral experience of families in the context of trisomy 13 and 18– a scoping review with thematic analysis

**DOI:** 10.1186/s12910-023-00994-x

**Published:** 2024-03-02

**Authors:** Zoe Ritchie, Gail Teachman, Randi Zlotnik Shaul, Maxwell J. Smith

**Affiliations:** 1 Faculty of Health Sciences and Rotman Institute of Philosophy, Western University, London, Ontario Canada; 2 School of Occupational Therapy, Faculty of Health Sciences, Western University, London, Ontario Canada; 3 Department of Paediatrics and Joint Centre for Bioethics, University of Toronto, Toronto, Ontario Canada; 4 Department of Bioethics , The Hospital for Sick Children, Toronto, Ontario Canada

**Keywords:** Bioethics, Decision-making, Goals of care, Values, Neonatal, Neonatal intensive care, Palliative care, trisomy 13, trisomy 18

## Abstract

**Introduction:**

The value of a short life characterized by disability has been hotly debated in the literature on fetal and neonatal outcomes.

**Methods:**

We conducted a scoping review to summarize the available empirical literature on the experiences of families in the context of trisomy 13 and 18 (T13/18) with subsequent thematic analysis of the 17 included articles.

**Findings:**

Themes constructed include (1) Pride as Resistance, (2) Negotiating Normalcy and (3) The Significance of Time.

**Interpretation:**

Our thematic analysis was guided by the moral experience framework conceived by Hunt and Carnevale (2011) in association with the VOICE (Views On Interdisciplinary Childhood Ethics) collaborative research group.

**Relevance:**

This article will be of interest and value to healthcare professionals and bioethicists who support families navigating the medically and ethically complex landscape of T13/18.

## Background

This paper adds to knowledge on the complexities of contemporary neonatal ethics discourse through a review and analysis of empirical literature concerning families’ moral experiences in the context of Trisomy 13 and 18 (T13/18). The value of a short life characterized by severe disability has been hotly debated in the literature on fetal and neonatal outcomes. Furthermore, in neonatal care, where decisions are made on behalf of a child, a challenge arises when we consider whose knowledge and values are considered authoritative in establishing goals of care and interventions in the best interest of a medically fragile infant. In some neonatal conditions, treatment options and anticipated outcomes are reasonably well known with a strong evidence basis in the medical literature. On the contrary, other conditions feature prognostic uncertainty that can make determining goals of care and appropriate medical intervention more uncertain and complex. Medical ethicist and physician John Lantos describes this as a distinct zone in neonatal ethics, which he refers to as the “stable grey zone” [1]. He describes three factors that would influence a condition and its associated treatment decision(s) to remain in this state of ambiguity: (a) if survival rates associated with a condition are critically low, (b) if survival rates are high but associated with severe neurocognitive deficiencies, and (c) if the treatment is long, complex, painful, and/or expensive [1]. In cases where the general public, the medical community and/or a patient group (or their substitute decision-makers) are divided on their confidence in either of these three factors, the condition is likely to remain in a *state of persistent ethical ambiguity*. Lantos offers trisomy 13 (Patau syndrome) and trisomy 18 (Edwards syndrome) as salient examples of conditions that exist in a state of persistent ethical ambiguity [1].

Ethical analysis in a state of persistent ethical ambiguity requires different evidence bases to evaluate how a divide in the perception of a condition has grown and persists between a broader public consensus, medical convention, and the experiences of a patient group.^1^ Our literature review is concerned with the evidence base of the latter– exploring the moral experiences of families^2^ in the context of T13/18. We do not contend understanding the moral experiences of families will bring the condition and its various treatment decisions out of a state of persistent ethical ambiguity. Rather, such understandings have the potential to illuminate the ‘invasiveness’ of this state. By invasiveness, we refer to how ethical ambiguity may coexist, influence, or be a barrier to decision-makers’ ability to realize the values they deem as critically important to making medical decisions. To our knowledge, no synthesis of the health-related literature on families’ experiences in the context of T13/18 has been performed to date. Our scoping review and subsequent thematic analysis seek: (a) to synthesize and analyze existing literature in terms of nature, features, volume, and (b) identify gaps in the existing pool of research.

### Trisomy 13 and 18

T13 and T18 belong to a class of chromosomal genetic disorders caused by an extra copy of the associated chromosome. After trisomy 21 (Down syndrome), these conditions are the second and third most commonly diagnosed autosomal trisomies in liveborn infants [2]. While T13 and T18 are distinct conditions, we have elected to include both in this review due to their similarities. This decision aligns with the broader health-related literature in the T13/18 area, where qualitative [3] and quantitative studies [4] often discuss the conditions in tandem.

A decade ago, T13/18 were largely considered conditions incompatible with life [5]. Research published within the last decade has demonstrated that 5–10% of infants with these conditions survive beyond their first year of life [2]. Children born with T13/18 have a combination of serious congenital disabilities. T13/18 are frequently (up to 80%) associated with multiple anomalies, including congenital heart defects, such as atrial or ventricular septal defect, patent ductus arteriosus, atrioventricular septal defect, tetralogy of Fallot, and intellectual disability [4]. Shifting perceptions of futility in cases of T13/18 have begun to change healthcare providers’ unwillingness to recommend life-prolonging measures, precipitating a recent upward trend in healthcare utilization among parents whose children have T13/18. A retrospective cohort study conducted in 2016 in Missouri found that between 1990 and 2014, major procedures on T13 patients increased from period 1 (1990–1997) to period 3 (2006–2013) from 0.11 to 0.78 procedures per patient [6]. For T18, the increase between the periods was from 0.14 to 1.33 procedures per patient [6]. The marked increases in these study results correspond with those reported in studies using national administrative data sets conducted outside of North America, including a 2015 study in Japan that measured medical procedures and outcomes of Japanese patients with T13/T18 [7]. These studies suggest changing perceptions on the futile nature of T13/18 may have significantly contributed to a substantial rise in the provision of major life-sustaining procedures on infants with T13/18.

### Moral experience

Our review adopts a moral experience framework developed for empirical and theoretical inquiry in bioethics by scholars Matthew Hunt and Franco Carnevale [8]. They define moral experience as *“[e]ncompassing a person’s sense that values that they deem important are being realized and thwarted in everyday life. This includes a person’s interpretations of a lived encounter, or a set of lived encounters, that fall on spectrums of right-wrong, good-bad, just-unjust”* ([8] p. 659). The framework focuses on five key elements: (a) *sense and interpretation*– specifying moral experience encompasses the ‘sense’ and ‘interpretation’ of particular encounters by individuals and the meanings they ascribe to particular experiences; (b) *the person*– specifying a focus on subjective experience with regard for the collective histories or contexts that constrain or shape an individual’s interpretation; (c) *values thwarted or realized in everyday life*– an individual’s sense that meaningful values are being enacted or impeded, highlighting the process of enacting or impeding values can occur simultaneously and in tension with one another; (d) *lived encounter or a set of lived encounters*– the sense that values are being realized or thwarted may or may not be linked to an identifiable person, object or circumstance and may include background orientations like a particular outlook on life; (e) *spectrums of right-wrong, good-bad, just-unjust*– describing spectrums, as opposed to categories, seeks to make visible variations and degrees of moral experience that can play out in both seemingly mundane and high pressure settings [8]. The framework was utilized in the development of the scope of our literature review and in the subsequent thematic analysis we pursued.

## Methods

### Identifying the research question and scope of the review

We used Arksey and O’Malley’s [9] methodological framework for conducting scoping reviews and heeded the enhanced recommendations of scholars Heather Colquhoun [10] and Danielle Levac [11]. We employed the MIP model for empirical literature reviews in bioethics to establish the scope of the review [12]. The question we produced to guide our review was, *“What is known in the literature about the moral experience of families navigating decision-making in the context of trisomy 13 or 18?”* Adopting a contextual framing in the review question is congruent with the method of analysis, a moral experience framework [8], which we subsequently employed in our thematic analysis, interpreting the idea of ‘context’ to include a wide range of phenomena, moral spectrums, and diverse circumstances.

### Identifying relevant studies

The literature we reviewed was retrieved through searches of the MEDLINE, EMBASE, CINHAL, and SCOPUS databases using a combination of search terms and keywords related to methodology (e.g., qualitative, interview, thematic), experiences (e.g., ethics, lived experience, decision-making), and participants (e.g., parents, pregnancy, trisomy 13, trisomy 18). We chose these databases to cover the broad range of disciplines scholars interested in the topic have published in (e.g., nursing, medicine, genetics, bioethics, social sciences). A general list of search terms was created and tested in each database. We further reviewed the reference list of included articles and conducted a grey literature review to enhance the rigour of the process and ensure no relevant articles were missed. We consulted with a research librarian at Western University, Meagan Stanley, throughout the design and execution of our search strategy. See Table 1 for terms used in the search strategy.

**Table 1:** Database Search Terms

Database	Search String
*Medline*	(((MH “Qualitative Studies”)) OR qualitative OR ((MH “Qualitative Studies”)) OR ((MH “Semi-Structured Interview”) OR (MH “Structured Interview”) OR (MH “Unstructured Interview”) OR (MH “Interviews”)) OR ((MH “Thematic Analysis”)) OR (“Mixed Method*”) OR “Interview*” OR “thematic” OR “theme*” OR narrative OR ((MH “Grounded Theory”)) OR (“Grounded Theory”) OR “observation” OR “ethnograph*” OR “phenomenolog*” OR ((MH “Ethnographic Research”)) OR ((MH “Phenomenology”))) AND ((((MH “Trisomy 13”)) OR ((MH “Trisomy 18”)) OR ((“trisomy 18” OR “edwards syndrome”)) OR ((“trisomy 13” OR “patau syndrome”))) AND ((((MH “Life Experiences”)) OR ((MH “Decision Making”)) OR ((MH “Palliative Care”)) OR ((MH “Quality of Life”)) OR ((MH “Genetic Screening”)) OR ((MH “Hope”)) OR ((MH “Motivation”)) OR ((MH “Parental Attitudes”) OR (MH “Parents”)) OR ((MH “Family”)) OR ((MH “Prenatal Care”) OR (MH “Prenatal Diagnosis”)) OR ((MH “Noninvasive Prenatal Testing”)) OR ((MH “Perinatal Care”)) OR ((MH “Infant Mortality”)) OR ((MH “Intensive Care Units, Neonatal”)) OR ((MH “Postnatal Care”)) OR ((MH “Ethics”) OR (MH “Ethics, Medical”) OR (MH “Ethics, Nursing”)) OR ((MH “Bioethics)))
*Embase*	(((MH “Qualitative Studies”)) OR qualitative OR ((MH “Qualitative Studies”)) OR ((MH “Semi-Structured Interview”) OR (MH “Structured Interview”) OR (MH “Unstructured Interview”) OR (MH “Interviews”)) OR ((MH “Thematic Analysis”)) OR (“Mixed Method*”) OR “Interview*” OR “thematic” OR “theme*” OR narrative OR ((MH “Grounded Theory”)) OR (“Grounded Theory”) OR “observation” OR “ethnograph*” OR “phenomenolog*” OR ((MH “Ethnographic Research”)) OR ((MH “Phenomenology”))) AND ((((MH “Trisomy 13”)) OR ((MH “Trisomy 18”)) OR ((“trisomy 18” OR “edwards syndrome”)) OR ((“trisomy 13” OR “patau syndrome”))) AND ((((MH “Life Experiences”)) OR ((MH “Decision Making”)) OR ((MH “Palliative Care”)) OR ((MH “Quality of Life”)) OR ((MH “Genetic Screening”)) OR ((MH “Hope”)) OR ((MH “Motivation”)) OR ((MH “Parental Attitudes”) OR (MH “Parents”)) OR ((MH “Family”)) OR ((MH “Prenatal Care”) OR (MH “Prenatal Diagnosis”)) OR ((MH “Noninvasive Prenatal Testing”)) OR ((MH “Perinatal Care”)) OR ((MH “Infant Mortality”)) OR ((MH “Intensive Care Units, Neonatal”)) OR ((MH “Postnatal Care”)) OR ((MH “Ethics”) OR (MH “Ethics, Medical”) OR (MH “Ethics, Nursing”)) OR ((MH “Bioethics)))
*CINHAL*	(((MH “Qualitative Studies”)) OR qualitative OR ((MH “Qualitative Studies”)) OR ((MH “Semi-Structured Interview”) OR (MH “Structured Interview”) OR (MH “Unstructured Interview”) OR (MH “Interviews”)) OR ((MH “Thematic Analysis”)) OR (“Mixed Method*”) OR “Interview*” OR “thematic” OR “theme*” OR narrative OR ((MH “Grounded Theory”)) OR (“Grounded Theory”) OR “observation” OR “ethnograph*” OR “phenomenolog*” OR ((MH “Ethnographic Research”)) OR ((MH “Phenomenology”))) AND ((((MH “Trisomy 13”)) OR ((MH “Trisomy 18”)) OR ((“trisomy 18” OR “edwards syndrome”)) OR ((“trisomy 13” OR “patau syndrome”))) AND ((((MH “Life Experiences”)) OR ((MH “Decision Making”)) OR ((MH “Palliative Care”)) OR ((MH “Quality of Life”)) OR ((MH “Genetic Screening”)) OR ((MH “Hope”)) OR ((MH “Motivation”)) OR ((MH “Parental Attitudes”) OR (MH “Parents”)) OR ((MH “Family”)) OR ((MH “Prenatal Care”) OR (MH “Prenatal Diagnosis”)) OR ((MH “Noninvasive Prenatal Testing”)) OR ((MH “Perinatal Care”)) OR ((MH “Infant Mortality”)) OR ((MH “Intensive Care Units, Neonatal”)) OR ((MH “Postnatal Care”)) OR ((MH “Ethics”) OR (MH “Ethics, Medical”) OR (MH “Ethics, Nursing”)) OR ((MH “Bioethics)))
*Scopus*	(TITLE ((trisomy 13) OR (trisomy 18) OR (edwards AND syndrome) OR (patau AND syndrome)) OR ABS ((trisomy 13) OR (trisomy 18) OR (edwards AND syndrome) OR (patau AND syndrome)) AND TITLE ((personal AND experience) OR (lived AND experience) OR (family AND coping) OR (decision AND making) OR (palliative AND care) OR (quality AND of AND life) OR (genetic AND testing) OR (moral AND experience) OR (medical AND intervention) OR (hope) OR (goal) OR (motivation) OR (parent*) OR (famil*) OR (prenatal) OR (perinatal) OR (neonatal) OR (postnatal) OR (ethic*) OR (bioethic*) OR (life AND experience*) OR (genetic AND screening) OR (infant AND mortality) OR (life AND change AND event*)) OR ABS ((personal AND experience) OR (lived AND experience) OR (family AND coping) OR (decision AND making) OR (palliative AND care) OR (quality AND of AND life) OR (genetic AND testing) OR (moral AND experience) OR (medical AND intervention) OR (hope) OR (goal) OR (motivation) OR (parent*) OR (famil*) OR (prenatal) OR (perinatal) OR (neonatal) OR (postnatal) OR (ethic*) OR (bioethic*) OR (life AND experience*) OR (genetic AND screening) OR (infant AND mortality) OR (life AND change AND event*)) AND TITLE ((qualitative) OR (mixed AND method*) OR (interview*) OR (thematic*) OR (theme*) OR (narrative) OR (grounded AND theory) OR (observation) OR (ethnograph*) OR (phenomenolog*)) OR ABS ((qualitative) OR (mixed AND method*) OR (interview*) OR (thematic*) OR (theme*) OR (narrative) OR (grounded AND theory) OR (observation) OR (ethnograph*) OR (phenomenolog*)))

### Study selection

**Fig. 1:**
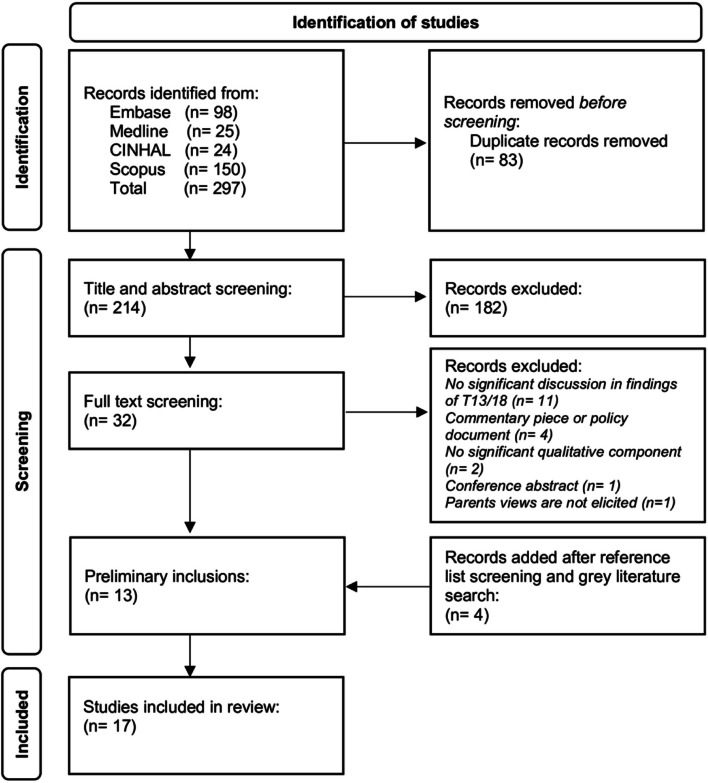
PRISMA Flow Diagram

Our study selection process began with developing inclusion and exclusion criteria (see Table 2). Our database search garnered 297 articles. We identified and removed 83 duplicates. The primary author performed the title and abstract screening of 214 articles, excluding 182 articles. A secondary reviewer from the authorship team (MS) worked with the primary author to review the remaining 32 full-text articles, excluding 19 articles. At this point, the two authors also searched grey literature and screened the reference lists of preliminarily included articles. After adding 4 articles from the grey literature search, this led to a total of 17 articles being selected for our review (see PRISMA diagram in Fig. 1).

**Table 2:** Inclusion and Exclusion Criteria

Inclusion Criteria	Exclusion Criteria
Study participants include parents of a child or a pregnant person (and their partner) who have received a trisomy 13 or 18 diagnosis during the prenatal or postnatal period of a pregnancy	No significant discussion in the findings of T13/18
Views on a parent’s or pregnant person’s (and/or their partner’s) experience in relation to their child (or pregnancy) are elicited	Commentary piece, policy document or conference abstract
Qualitative methods or mixed methods (with a significant reported qualitative component) are used	No significant qualitative component
Study is published in a peer-reviewed journal	Study is not available in English

### Charting the data

We extracted descriptive data (See Table 3) from articles, including the geographical study location, disciplinary location of the primary author (if more than two authors were listed the first was considered primary), research question/topic, type(s) of qualitative methods used, data analysis of the qualitative component, makeup of participants, and the key findings of the qualitative component.

**Table 3:** Descriptive Data of Included Articles

**Article Reference #1**	
Agatisa P.K., et al., (2015). A first look at women’s perspectives on noninvasive prenatal testing to detect sex chromosome aneuploidies and microdeletion syndromes [13].	
**Descriptive Details**	
*Study country took place*	United States
*Disciplinary location of primary author(s)*	Medicine (Agatisa, P.)
*Research question/topic*	The aim of this study is to explore women’s opinions about the use of noninvasive prenatal testing (NIPT) to assess the risk of sex chromosome aneuploidies and microdeletion syndromes.
*Type(s) of qualitative methods used*	Focus groups
*Data analysis of qualitative component*	Interpretive Description
*Participants*	Women who were either pregnant or recently delivered (18 to 45 years of age) recruited from a sample of 68 women who had taken part in a previous study about the use of NIPT as a screening tool for common autosomal aneuploidies including T21, 18, and 13.
**Key findings of Qualitative Component**	
1) Familiarity with conditions being assessed by non-invasive prenatal testing (NIPT) affected patients’ perceptions of the value of testing. 2) Participants in this study were well versed in the use of prenatal testing to assess the presence of Trisomies 21, 18, and 13, the utility of NIPT as a screening test, and the need for diagnostic testing to confirm the presence or absence of a condition. 3) Patients supported the ability to opt-in or opt-out of testing for certain conditions based on the value they assign to acquiring such information. 4) Patients trusted their obstetric clinicians and relied on them to provide the information required to make informed decisions about NIPT. 5) Patients recognized that clinicians have limited time and resources for patient education and counselling in addition to their responsibility as patients to be informed healthcare consumers.	
**Article Reference #2**	
Arthur, J., Gupta, D. (2017). “You Can Carry the Torch Now:” A Qualitative Analysis of Parents’ Experiences Caring for a Child with Trisomy 13 or 18 [3].	
**Descriptive Details**	
*Study country took place*	United States
*Disciplinary location of primary author(s)*	Medicine (Arthur, J.) Medicine (Gupta, D.)
*Research question/topic*	This study sought to interview experienced families to provide accounts of what a parent might expect when caring for a child with Trisomy 13/18.
*Type(s) of qualitative methods used*	Interviews
*Data analysis of qualitative component*	Grounded Theory
*Participants*	Families (parents) of children with diagnoses of T13/18.
**Key findings of Qualitative Component**	
1) Parents saw their children as significant, having special importance within their communities and faith traditions. As a result, they resisted attempts to reduce their child to a medical diagnosis. 2) Parents saw their child as having a transformative effect on those around them. However, while the effects were often positive, parents warned that the challenges of their child’s complex disease could also create ruptures in relationships. 3) Parents felt that engaging in the medical system brought feelings of powerlessness which alienated them from their child. 4) Parents emerged from these experiences motivated to tell their child’s story, believing that storytelling could be therapeutic for them and helpful for others.	
**Article Reference #3**	
Côté-Arsenault, D., & Denney-Koelsch, E. (2011). “My Baby Is a Person”: Parents’ Experiences with Life-Threatening Fetal Diagnosis. Journal of Palliative Medicine [14].	
**Descriptive Details**	
*Study country took place*	United States
*Disciplinary location of primary author(s)*	Nursing (Côté-Arsenault, D.) Medicine (Denney-Koelsch, E)
*Research question/topic*	This study sought to clarify the experiences and needs of families in order to design responsive perinatal palliative care services, and to establish the feasibility and acceptability of conducting intensive interviews of pregnant women and their partners during their pregnancy with a life-threatening fetal diagnosis.
*Type(s) of qualitative methods used*	Interviews
*Data analysis of qualitative component*	Thematic Analysis
*Participants*	Women and couples who were pregnant with a baby with a postbirth life expectancy of 2 months or less and had plans to continue the pregnancy.
**Key findings of Qualitative Component**	
1) Parents grieved the loss of their normal pregnancy, healthy baby, and future. 2) Parents experienced a sudden interruption in the normal process of becoming a parent. 3) Parents held a unanimous desire to honor and legitimize the humanity of their unborn baby. 4) Parents experienced disjointed and distant encounters with multiple providers. 5) Parents described a lack of understanding from family and friends of what they were experiencing. 6) Parents’ sense of social isolation added to their personal sense of loss and loneliness.	
**Article Reference #4**	
Guon, J., et al., (2014). Our children are not a diagnosis: The experience of parents who continue their pregnancy after a prenatal diagnosis of trisomy 13 or 18 [5].	
**Descriptive Details**	
*Study country took place*	United States
*Disciplinary location of primary author(s)*	Law/Bioethics (Guon, J.)
*Research question/topic*	The study asked parents who belong to T13 (and/or) 18 internet support groups about their prenatal experience, their hopes, the life of their affected child, and their family experience. This article is part of a larger study; for the purpose of this article the analysis was focused on a subgroup of parents who received a prenatal diagnosis.
*Type(s) of qualitative methods used*	Survey (with open ended questions)
*Data analysis of qualitative component*	Thematic Content Analysis
*Participants*	Parents of children with T13/18 who were part of online social support networks and received a prenatal diagnosis.
**Key findings of Qualitative Component**	
1) Parents who received a prenatal diagnosis of T13/18 expressed reasoning related to moral beliefs (personal values and/or religious beliefs), parent centered reasons (desires or perceived benefits of the pregnancy/child) and child centered reasons (love, intrinsic value, uncertain outcome). 2) Parents felt some health care providers did not view children with T13/18 as unique children, though over half reported positive experiences with a “special” provider who provided balanced information and treated them with respect. 3) Parents reported feeling pressure to terminate the pregnancy. 4) Parents described their hopes when they first heard about the diagnosis, the most common being that the child would be born alive and they would have a modest amount of time to spend with their child. 5) The majority of parents whose child died described the overall experience of their child’s life as being positive, irrespective of the length of their lives. 6) The authors offer suggestions to assist healthcare providers to provide optimal prenatal care for parents who continue their pregnancy after a diagnosis of T13/18.	
**Article Reference #5**	
Janvier A. et al., (2016). Parental hopes, interventions, and survival of neonates with trisomy 13 and trisomy 18 [15].	
**Descriptive Details**	
*Study country took place*	United States
*Disciplinary location of primary author(s)*	Medicine/Bioethics (Janvier)
*Research question/topic*	This article is part of a larger study to describe the experiences of parents who are members of social networks and who have (had) children with T13/18. The objective of this article was to examine parental goals and hopes after a T13/18 diagnosis and to provide practical recommendations and behaviors clinicians could emulate to avoid conflict.
*Type(s) of qualitative methods used*	Survey (with open ended questions)
*Data analysis of qualitative component*	Thematic Content Analysis
*Participants*	Parents of children with T13/18 (full/complete) who were part of online social support networks.
**Key findings of Qualitative Component**	
1) Parents had common hopes when they received a diagnosis of T13/18- they hoped to meet their child alive, take their child home, be a family and give their child a good life. 2) The recommendations parents had from medical providers were homogeneous; comfort care at birth with the plan of not prolonging life was recommended to all parents 3) The single most important factor independently related to mortality (before going home or before 1 year) even when correcting for all other factors, was the presence of a prenatal diagnosis. 4) The authors found that palliative care was homogeneous (minimal interventions and no interventions to prolong life) for children with a prenatal diagnosis. On the other hand, palliative care after a postnatal diagnosis seemed more individualized to the child’s needs and the family’s decisions. 5) Decisions were influenced by the state of the child and whether they were vigorous or weak with parents in general not wanting to impose undue suffering. Parents of almost half the children discharged on comfort care later decided to consider surgical interventions, because their child exceeded expectations.	
**Article Reference #6**	
Janvier, A. et al., (2012). The Experience of Families With Children With Trisomy 13 and 18 in Social Networks [16].	
**Descriptive Details**	
*Study country took place*	United States
*Disciplinary location of primary author(s)*	Medicine/Bioethics (Janvier)
*Research question/topic*	The overall objective of this study was to describe the experiences of parents who are members of social networks and who have (had) children with T13/18.
*Type(s) of qualitative methods used*	Survey (with open ended questions)
*Data analysis of qualitative component*	Thematic Content Analysis
*Participants*	Parents of children with T13/18 who were part of online social support networks.
**Key findings of Qualitative Component**	
1) Of the parents whose children died the vast majority reported that the overall experience of their child’s life was positive. 2) Just under half of parents whose child lived longer than one year found the financial sacrifices related to their child to be very challenging. 3) Parents reported appreciating healthcare providers who- referred to the child by name (even if unborn), offered to take pictures (in and ex utero), referred to other families or web sites, and described not only those organs that had malformations but also those that did not have malformations. 4) The most common negative comment made by parents was a sense that health care providers did not see their baby as having value, as being unique, as being a baby.	
**Article Reference #7**	
Janvier, A. et al., (2020). Building trust and improving communication with parents of children with Trisomy 13 and 18: A mixed-methods study [17].	
**Descriptive Details**	
*Study country took place*	United States
*Disciplinary location of primary author(s)*	Medicine/Bioethics (Janvier)
*Research question/topic*	This article is part of a larger study to describe the experiences of parents who are members of social networks and who have (had) children with T13/18. The objective of this article was to examine parents prenatal experience, their hopes, the life of their affected child, and their family experience. The analysis focused on survey questions related to communication between clinicians and parents.
*Type(s) of qualitative methods used*	Survey (with open ended questions)
*Data analysis of qualitative component*	Thematic Content Analysis
*Participants*	Parents of children with T13/18 who were part of online social support networks.
**Key findings of Qualitative Component**	
1) Parents described trust as central to positive and supportive interaction with clinicians. 2) Knowledge functioned as an element of trust– The ability of clinicians to give general as well as personalized information, to be humble and curious and to give balanced information functioned as elements of trust. 3) Caring and valuing the child and the family functioned as an element of trust– parents reported positive clinicians cared about them and made them feel their baby and their family had value, were kind and made them feel like good parents. 4) Support and hope functioned as an element of trust– knowing somebody would be there with them during difficult times/decisions, advocate for their child and provide hope. 5) Parents descriptions of disagreements and conflicts involved vulnerability and uncertainty (lack of knowledge and ambivalence toward decisions), distrust (parents felt that clinicians thought their child was “better off dead,” had no value) and anger or certainty (parents demanded second opinions or active interventions). 6) When difficult interactions were described by parents a significant number felt pressure regarding end-of-life decisions or decisions about medical interventions.	
**Article Reference #8**	
Kosho, T. et al., (2013). Natural history and parental experience of children with trisomy 18 based on a questionnaire given to a Japanese trisomy 18 parental support group [18].	
**Descriptive Details**	
*Study country took place*	Japan
*Disciplinary location of primary author(s)*	Medicine/ Genetics (Kosho)
*Research question/topic*	This study sought to collect detailed clinical information of children with T18 and parental experiences in order to contribute to a more comprehensive characterization of children with T18.
*Type(s) of qualitative methods used*	Survey (with open ended questions)
*Data analysis of qualitative component*	No specific method of analysis reported
*Participants*	Parents of a children with T18 (full/complete) who belonged to an online T18 support group. The open-ended questions excluded parents whose child did not survive for one day.
**Key findings of Qualitative Component**	
1) Parents appeared to be positive about caring for their children and the children seemed to interact with parents and siblings for as long as they lived which resulted in quality time and happiness within the families. 2) The most common issue that the parents found difficult was the physical condition of their children and related medical care (exhaustion from home medical care and anxiety for the future of their children).	
**Article Reference #9**	
Lebedoff A.N. & Carey J.C. (2021). Parent-reported histories of adults with trisomy 13 syndrome [19].	
**Descriptive Details**	
*Study country took place*	United States
*Disciplinary location of primary author(s)*	Medicine/ Genetics (Lebedoff A.N & Carey J.C.)
*Research question/topic*	The goal of this study was to collect the medical histories of adult individuals with apparent non-mosaic T13 to help gain further insight into the clinical course for individuals with this condition and to characterize the manifestations for surveillance and management.
*Type(s) of qualitative methods used*	Interviews
*Data analysis of qualitative component*	No specific method of analysis reported
*Participants*	Parents representing adult individuals with apparent non-mosaic T13 by parental report, or verified by clinical chromosome testing or clinical documentation when available.
**Key findings of Qualitative Component**	
1) The authors provide a list of comments from family members on the question of what they would like a family to know who has a new diagnosis of T13 in their infant. These quotes describe a diversity of emotions and sentiments including fear, grief, joy, patience, growth, familial fit, adjustment periods and challenges of living in a rural area. They all had a generally hopeful or positive tone.	
**Article Reference #10**	
Lewis C., et al., (2016). A qualitative study looking at informed choice in the context of non-invasive prenatal testing for aneuploidy [20].	
**Descriptive Details**	
*Study country took place*	England and Scotland
*Disciplinary location of primary author(s)*	Psychology (Behavioural Science)
*Research question/topic*	To explore women’s attitudes towards non-invasive prenatal testing (NIPT) and determine factors influencing their decisions around uptake of NIPT.
*Type(s) of qualitative methods used*	Interviews
*Data analysis of qualitative component*	Thematic analysis
*Participants*	Women who booked screening before 20 weeks gestation who accepted down syndrome screening as part of routine care.
**Key findings of Qualitative Component**	
1) The majority of women were aware that NIPT tested for trisomy 13 and 18, in addition to Down syndrome, but interviews highlighted that most participants had no knowledge of these conditions prior to being informed about them either at the booking-in-appointment or when they were offered NIPT. Most women recollected that these conditions were much more severe than Down syndrome. 2) The authors found three dominant factors shaped women’s attitudes toward NIPT a) Women found results were easier to understand and more accurate than traditional screening making NIPT feel reassuring and safe b) Women’s perceptions of whether they felt physically and mentally able to take on the challenges of caring for a disabled child were found to play a significant role in influencing their attitudes towards undergoing NIPT c) Moral or religious views around termination of pregnancy and perceived quality of life of a child with Down syndrome (or T13/18) were frequently cited when women reflected on their attitudes towards NIPT. 3) Most women agreed that the quality of life of a child with T13/18 was so poor that it did justify termination of pregnancy.	
**Article Reference #11**	
Park A & Mathews M. (2009). Women’s decisions about maternal serum screening testing: A qualitative study exploring what they learn and the role prenatal care providers play [21].	
**Descriptive Details**	
*Study country took place*	Canada
*Disciplinary location of primary author(s)*	Park, A. (undetermined) Mathews, M. (Medicine/ Health Humanities)
*Research question/topic*	The study investigates how well-informed women’s screening decisions are, what and from which sources do women learn maternal serum screening and what role prenatal care providers play in these decisions.
*Type(s) of qualitative methods used*	Interviews
*Data analysis of qualitative component*	Thematic Analysis
*Participants*	Pregnant women in their 21-26th week of pregnancy.
**Key findings of Qualitative Component**	
1) Women in the study relied upon multiple sources of information to learn about MSS including healthcare professionals, prenatal pamphlets, books, the internet, on-line chat rooms, prenatal classes and family and friends. The manner in which women in the study learned about MSS (use of multiple sources, reliance on personal connections) was consistent with the way they learned about other aspects of their pregnancy. 2) Many women felt after speaking to their physician about MSS they needed additional information to make their decisions and identified special topics that were not discussed (how the test is performed, accuracy, emotional preparation). 3) The way physicians presented information about the test influenced women’s attitudes regarding the value of the test.	
**Article Reference #12**	
Reinsch, S. et al., (2021). Decision-making about non-invasive prenatal testing: Women’s moral reasoning in the absence of a risk of miscarriage in Germany [22].	
**Descriptive Details**	
*Study country took place*	Germany
*Disciplinary location of primary author(s)*	Medicine
*Research question/topic*	This study investigates women’s (and some of their partners’) experiences with, and practices of decision-making about, non-invasive prenatal testing (NIPT).
*Type(s) of qualitative methods used*	Interviews
*Data analysis of qualitative component*	No specific method of analysis reported
*Participants*	Women who had previously used or declined NIPT (and their partners).
**Key findings of Qualitative Component**	
1) Women who decided for NIPT, were often motivated by its offer of clarity and reassurance for different aims (e.g., to terminate the pregnancy in case of a positive test result, to confirm everything was fine with their pregnancy or to prepare for the birth of and life with a disabled child). 2) Women who made use of NIPT reported that because of the lack of risk, they did not seriously reflect upon the decision of whether to take NIPT as an additional test. 3) Dilemmas within social relations emerged in the context of NIPT (e.g., problems with differing “risk evaluations” between physicians, women, and their partners and existential troubles that emerge in families that already include people with a disability, particularly when woman contemplated taking a test that might lead to the abortion of a disabled fetus).	
**Article Reference #13**	
Snure Beckman, E. et al., (2019). Attitudes Toward Hypothetical Uses of Gene-Editing Technologies in Parents of People with Autosomal Aneuploidies [23].	
**Descriptive Details**	
*Study country took place*	United States
*Disciplinary location of primary author(s)*	Genetics
*Research question/topic*	The authors sought to investigate the attitudes of parents of children with T21/18/13 toward hypothetical uses of gene-editing technologies for their children and others.
*Type(s) of qualitative methods used*	Interviews
*Data analysis of qualitative component*	No specific method of analysis reported
*Participants*	Parents of people with a T13, 18 or 21 diagnoses
**Key findings of Qualitative Component**	
1) Parents of people with T21 differed from parents of people with T13/T18 in how they envisioned gene editing impacting their child. Most parents of people with T21 reported that their children were minorly impacted by T21-associated health issues, but if their child’s health issues were more significant, it would impact their willingness to use gene editing. 2) Parents desired increased oversight and guidelines as gene-editing technologies become more clinically feasible. 3) Among those who might use gene editing for their children, primary motivations centered around improving quality of life. The exact mechanism by which they desired to improve quality of life varied among participant populations. Parents of people with T18/13 focused on ameliorating life-threatening health issues, while parents of T21 emphasized increasing their children’s communication and cognitive ability in order to increase independence. 4) An overarching concern was that such technologies would eventually eliminate disability from society and that their children bring a unique perspective to the world that does not need to be “fixed.”	
**Article Reference #14**	
Walker LV, Miller VJ, & Dalton VK. (2008). The health-care experiences of families given the prenatal diagnosis of trisomy 18 [24].	
**Descriptive Details**	
*Study country took place*	United States
*Disciplinary location of primary author(s)*	Genetics
*Research question/topic*	This study explores families’ overall experiences in the health care system after receiving a diagnosis of trisomy 18. The objective of this study was to examine the quality of their interaction with health care providers and to identify aspects of care associated with satisfaction.
*Type(s) of qualitative methods used*	Interviews
*Data analysis of qualitative component*	Classical Content Analysis
*Participants*	Women (and their partners) who received a diagnosis of T18 prenatally since 2000.
**Key findings of Qualitative Component**	
1) Parents expressed positive patient-provider relationships from their perspective involved the expression of empathy and compassion. They also valued providers who treated the fetus as valuable and the pregnancy like any other. 2) When discussing negative patient-provider relationships parents found discontinuity of care, poor communication, lack of information/education on the screening process, and generally poor delivery of results, to negatively impact their experiences. 3) In recalling their (parents) experience of receiving the result, prognostic information, and options-counselling some parents were sensitive to language used (incompatible with life, no hope) while others appreciated a perceived straightforwardness to the language. 4) Several families described having to ‘fight’ on behalf of their fetus. Families desired autonomy over the decision-making process and many chose not to seek counsel from providers who seemed biased toward certain options. Some families reported that they feared their fetus would receive substandard care from providers not interested in supporting their hopes and preferences for the remainder of the pregnancy and delivery.	
**Article Reference #15**	
Wallace, S. E. et al., (2018). Parent Perspectives of Support Received from Physicians and/or Genetic Counselors Following a Decision to Continue a Pregnancy with a Prenatal Diagnosis of Trisomy 13/18 [25].	
**Descriptive Details**	
*Study country took place*	United States
*Disciplinary location of primary author(s)*	Medicine/Genetics
*Research question/topic*	This study investigated the extent to which families that choose to continue a pregnancy with a prenatal diagnosis of Trisomy 13/18 felt supported by their healthcare providers, and any differences in the perceived level of support experienced by those working with a physician versus those working with a genetic counsellor.
*Type(s) of qualitative methods used*	Survey (with open ended questions)
*Data analysis of qualitative component*	No specific method of analysis reported
*Participants*	Families who belong to an online support group who had or have a child with a trisomy diagnosis.
**Key findings of Qualitative Component**	
1) When comparing the communication styles of genetic counsellors and physicians, this study found that parents generally perceived genetic counsellors as better able to convey the necessary information objectively and in an understandable way. 2) Less than half of all respondents felt that genetic counsellors and physicians provided them with all of the information needed to make an informed decision. Very few parents reported being told that there was a possibility of their child living past birth. This piece of information could be crucial for those that are considering termination or continuation.	
**Article Reference #16**	
Weaver, et al., (2020). Mixed method study of quality of life for children with trisomy 18 and 13 after cardiac surgery [26].	
**Descriptive Details**	
*Study country took place*	United States
*Disciplinary location of primary author(s)*	Medicine (Weaver)
*Research question/topic*	The study explored the explored the experiences, hopes, understanding, and goals of parents of a child with trisomy 18 who made the decision to access cardiac interventions after the birth of their child.
*Type(s) of qualitative methods used*	Interviews
*Data analysis of qualitative component*	Content Analysis
*Participants*	Mothers of children with a T18 diagnosis accessing cardiac interventions for their child after birth.
**Key findings of Qualitative Component**	
1) Mothers were told by healthcare providers following their child’s diagnosis to prepare to lose their baby and expressed frustration over the dominance of that narrative in their discussions. 2) Mothers expressed diverse hopes, including– for their child to enhance future options for children with their condition, to be treated like the unique child they are, and to be able to spend quality time with their child. 3) When mothers discussed their understanding of their child’s prognosis they balanced discussing specific medical information with expressions of uncertainty. 4) In discussing the attitudes of providers who did not agree with offering aggressive medical or surgical interventions to infants with trisomy 18 mothers expressed they wished to be treated with compassion, a respect for their hopes and goals for their child, and an acknowledgment of the value in creating environments that encourage listening and openness.	
**Article Reference #17**	
Weaver. et al., (2018). Eliciting Narratives to Inform Care for Infants With Trisomy 18 [27].	
**Descriptive Details**	
*Study country took place*	United States
*Disciplinary location of primary author(s)*	Medicine
*Research question/topic*	The primary objective of this study was to quantify perception of child and parent quality of life and family impact for children with trisomy 18 or trisomy 13 who had undergone definitive cardiac surgery. The study further aimed to explore the child’s functional status as assessed by parents and health care providers and to correlate functional status with family impact. The study described the child’s quality of life from parental qualitative narratives, the family’s experience accessing surgical interventions, parental hopes for their child, and parental advice for medical teams.
*Type(s) of qualitative methods used*	Survey (with open ended questions)
*Data analysis of qualitative component*	Semantic Content Analysis
*Participants*	Parents of children with T18/T13 who had travelled out of state to access cardiac surgical interventions denied to them in their local care setting due to genetic diagnoses.
**Key findings of Qualitative Component**	
1) The quality of life, impact of the child’s diagnosis and prognosis, and a family’s function and well-being were subjective and differed from family to family. The children in this study were perceived by their families to have high quality of life and valued life roles. 2) The functional status of the children was noted to be congruent between parent and provider perspectives. Functional status had minimal impact on the parental perception of the child’s quality of life or parental perception of overall family wellness. 3) Parents depicted their hopes for their child in terms of allowing the child to reach his or her unique potential, experiencing joy and happiness, knowing love, inspiring others, having access to medical care in the future, and finding comfort in the family’s spiritual framework.	

### Descriptive features of included articles

The majority of studies we included in the review were conducted in the United States [13], with the remainder having diverse disciplinary locations, including Japan [1], England/Scotland [1], Canada [1] and Germany [1]. The disciplinary backgrounds of the primary authors were also diverse, including Medicine, Bioethics, Nursing, Genetics, Psychology, Law, and Health Humanities. All articles were published in the last 13 years, with the oldest published in 2008 [24] and the most recent in 2021 [15, 19]. Studies examined different experiences in the context of T13/18, including screening in the prenatal phase, experiences during the pregnancy after diagnosis, and experiences of families after birth. Interviews and focus groups were the most popular data collection method (58%), followed by surveys with open-ended questions (41%). A significant number of studies that reported a method of analysis for the qualitative component of their study used a form of thematic analysis (58%) or content analysis (23%). Other methods of qualitative analysis included interpretive description and grounded theory. A significant number of studies (41%) did not report a specific method for the analysis of their qualitative component, though most did organize the findings into ‘themes’. Four articles [5, 15–17] are associated with a larger study [16]. Despite similarities in data reported, we included all four articles as they produced distinct analyses. No studies included in the review used the term moral experience or applied it as a framework [8].

### Data analysis

**Fig. 2:**
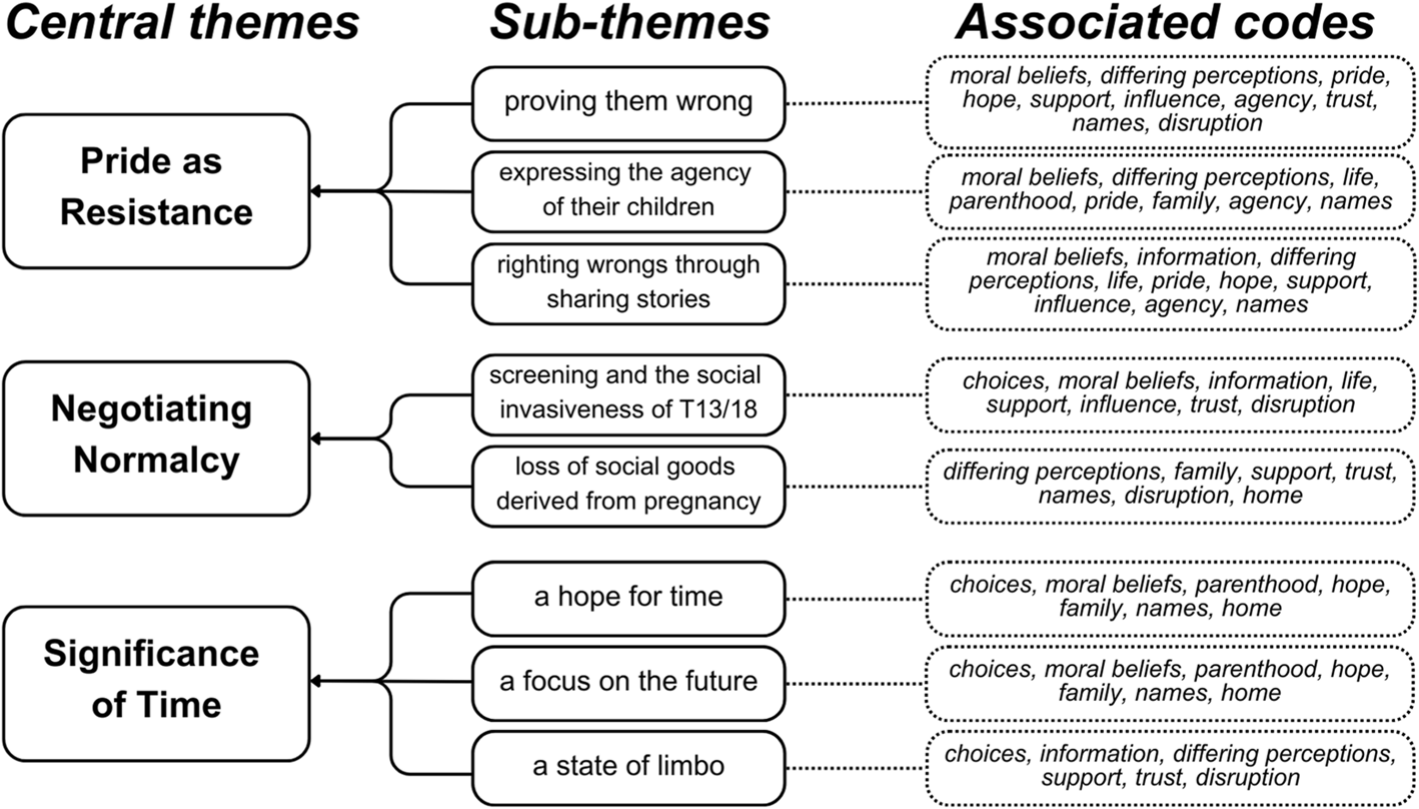
Thematic Map

We employed reflexive thematic analysis proposed by scholars Braun and Clarke [28] to analyze themes across articles in our review. The first step involved close reading and note-taking to begin to think about codes. We moved between articles to begin to appreciate patterns among them. We coded textual excerpts of quotes from participants in the studies reviewed and interpretations from the authors of the studies. Quirkos [29], a qualitative data management software, was used to organize the data, and all coding was performed manually. A deductive coding scheme (See Table 4) was then used as the analytic interests in the review were informed by Hunt and Carnevale’s moral experience framework [8]. As none of the studies explicitly used childhood ethics language, we focused on producing codes that would seek to broadly interpret the features of the moral experience of families as expressed across the studies included in this review. Several codes (e.g., choices, moral beliefs, life, family) are purposively broad. These codes were further developed into sub-themes and often a code was applied to multiple sub-themes. Our analysis resulted in three distinct themes: (a) Pride as Resistance, (b) Negotiating Normalcy, and (c) The Significance of Time. Themes are described below, with accompanying excerpts from the studies included in this review. See Fig. 2 for a thematic map reflecting the relationship between codes, sub-themes, and central themes.

**Table 4:** Coding Scheme

Codes	Definitions
*Choices*	Discussions or references to barriers or facilitators to making decisions between a range of choices.
*Moral Beliefs*	Discussions of beliefs or wishes that have a connection to an individual’s sense of good/bad, just/injustice, fair/unfairness.
*Information*	How information is shared, learned, delivered, or represented.
*Differing Perceptions*	Discussions or references to parties differing on a way of regarding, understanding, or interpreting something significant.
*Life*	Discussions or references to what makes a life valuable.
*Parenthood*	Discussions or references to what it means to be a good/bad parent.
*Family*	Discussions or references to the significance of family.
*Support*	Discussions or references to supportive actions or expressions.
*Influence*	Discussions of parties’ capacity to influence the character, development, or behaviors of someone or something.
*Agency*	Observed expressions of agency (action or intervention, especially such as to produce a particular effect).
*Trust*	Discussions of references to beliefs in the reliability, truth, ability, or strength of someone or something.
*Names*	References to the importance of using the names of individuals.
*Pride*	Discussions where parents express pride in their child or parenting.
*Disruption*	Discussions of circumstances where individuals express a disruption to meaningful aspects of their life.
*Hope*	Discussions or references to hope.
*Home*	Discussions or references to the significance of being with their child at home, or bringing their child home.

### Findings

#### Pride as resistance

Across multiple studies, parents of infants with T13/18 who chose to continue their pregnancy to term reported facing significant opposition from healthcare professionals who questioned whether their choices were in the best interest of their child or were primarily concerned with self-interests. Several scholars suggested it was common for parents who chose to continue their pregnancy after a T13/18 diagnosis to face significant, strong opposition from healthcare professionals.

*“The doctor blurted out she was going to die before everyone left the room, even my 4-year-old son.” (study participant, Janvier et al.,* [15]*, p. 282).*

*“When I heard a nurse say to another one “She must be so uncaring and unloving making that baby suffer!” A nurse told us “We want what is in her best interests. Why would you want that done to your child?” (study participant, Janvier et al.,* [17]*, p. 266).*

This sentiment was expressed by several study participants, like those above, across articles in our review. Further, researchers in multiple studies underscored the prominence of this sentiment.

*“Following the diagnosis, parents report being told by a HCP that their baby would likely die before or at the time of birth (94%), that their baby would not live for more than a few months (88%), that the condition of their baby was lethal or incompatible with life (93%), that their child would be a vegetable (55%), that their baby would destroy their family or their marriage (28%), and that if their baby survived, he would live a meaningless life (55%) or a life of suffering (68%).” (Guon et al.,* [5]*, p. 312).*

*“Most parents had been told their child was “incompatible with life” and would live a meaningless life of suffering (Guon et al.,* [5]*)* but perhaps experienced the opposite; their baby grew, smiled, and progressed and the family coped and was enriched. A quarter of parents regretted not doing enough interventions.” (Janvier et al., [15], *p. 285).*

Although many parents reported finding this opposition distressing, multiple studies in our review suggested it may have contributed to the development of resiliency that became significant to their ability to navigate the psychosocial challenges of their clinical and personal environments. Both study participants in the description of their experiences, and researchers in the presentation of findings, expressed this resistance through fighting metaphors.

*“Several families described having to fight on behalf of their fetus.” (Walker* et al.*, *[24]*, p. 17).*

*“I think that healthcare providers are so negative about the baby they force families on the defensive […] If we feel providers want our babies dead, we will fight for them. This is what parents do.” (study participant, Janvier et al.,* [17]*, p. 267).*

*“My understanding of prognosis is that the odds are stacked against my daughter. She has got a lot of uphill battles to fight.” (study participant, Weaver et al.,* [27]*, p. 2).*

In comparison, other studies presented the experiences of parents who expressed that developing and maintaining this resiliency within clinician environments often led to severe exhaustion and frustration.

*“We did not know what to do. Then I got pressure to terminate my pregnancy. They told me she would ruin my life and there was no hope. This was too much. My baby was worth it and I would get what she needed. We went somewhere else.” (study participant, Janvier et al.,* [17]*, p. 267).*

*“The narrative impulse (of participants) seemed to be a response to their feelings of powerlessness as they struggled to grasp their child’s diagnosis. They described feeling at odds with the medical staff.” (Arthur and Gupta,* [3]*, p. 235).*

*“We were unsure of what to do about surgery, this was our child’s life […] they (medical team) were sure it would be better if he died quickly. It seemed very simple to them. When they told us there was nothing they could do, and all the things he would not get, we decided this was enough and that we would get something, everything for our baby.” (study participant,* *Janvier* et al., [17], *p. 267)*.

In multiple studies, researchers presented the experience of parents who advocated for their child’s agency, personhood, and unique attributes to be recognized by individuals around them.

*“She had a peaceful character, a sense of humor, a toughness that always made her try hard and never give up, and friendliness.” (study participant, Kosho et al.,* [18]*, p. 1538).*

*“She loves to ride her horse with her special saddle and support, pet her cats, and pet her dogs. She loves to play in the water or float and splash. She is the light of our lives. She is almost always happy and she’s full of sass. She is the strongest person I know and I’m so proud to be her mom.” (study participant, Weaver et al.,* [26]*, p. 234).*

*“All of the parents interviewed described unique and distinctive qualities for their child. They often characterized their child as energetic and opinionated, using words like “bossy” and “magnetic” (#10), “feisty,” and “proud of herself” (#17), “spunky” and “sassy” (#28), and a “princess warrior” that ran her household (#10).” (Arthur and Gupta,* [3]*, p. 228).*

*“Some parents saw their child as an autonomous entity with his or her own agency and plan. One family felt encouraged by their doctor telling them that their daughter would “tell her own story.” (Arthur & Gupta,* [3]*, p. 228).*

Studies also reported the parents they interviewed found value in like-minded communities who had lived experience in advocating for their infant with T13/18 and navigating the medical system. Storytelling within these communities emerged as a meaningful interest for families.

*“All of the families expressed a particular interest in supporting other families who had children with T 13/18. One mother described her daughter as a 1-year-old with a desire to “impact so many people,” a “purpose” that her mother perceived her daughter passing to her in her final hours: “Mom, I am tired, you can carry the torch now.” (Arthur & Gupta,* [3]*, p. 233).*

*“If there is something that we can say from our experience that will help support somebody else, that’s really important because we want her life to have purpose and we want her life to have meaning. And anything that we can do to make sure that stays forever I’ll do (#12).” (study participant, Arthur & Gupta,* [3]*, p. 233).*

#### Negotiating normalcy

A subset of articles in this review focused on family screening and pregnancy experiences in the context of T13/18. Several researchers found that while the screening process for T13/18 commonly involves noninvasive medical testing (did not require surgical intervention), this testing had intrusive implications for parents’ lives in another way, as highly socially invasive.

*“One prenatal diagnostician made a powerful claim concerning the social implications of NIPT when she said that “I know it calls itself a noninvasive test, but basically, it is a highly invasive test. For me, this is an invasive test, though not with a needle, yet it is highly intrusive.” In line with this claim, we argue that despite its clinical non-invasiveness, NIPT affects decision-making and is in this sense socially invasive. It can introduce disturbances into, and interferences with, social relationships.” (Reinsch et al.,* [22]*, p. 210).*

Researchers found that some parents interviewed pursued screening for the perceived feelings of reassurance they believed a negative test could offer the rest of their pregnancy experience.

*“NIPT, in contrast was frequently described as reassuring […] for the majority of women NIPT enabled them to have reassurance that the baby was not affected by one of the three main trisomies and reduced anxiety for the remainder of the pregnancy.” (Lewis et al.,* [20]*, 877).*

Conversely, other researchers found some parents expressed the anticipated distress and burden to their psychological health, in the event of a positive test, outweighed possible benefits the knowledge of the test might have offered them.

*“In our study, we found that some women chose the strategy of non-testing in order to avoid the psychological distress and burden they expected to be caused by NIPT. For these women, the experience of an unburdened pregnancy outweighed the knowledge that the test provides.” (Reinsch et al.,* [22]*, p. 211).*

One researcher [22] described how prenatal testing influenced how parents morally positioned themselves toward their fetus and other people in their lives with disabilities.

*“not only [did she] position herself towards the fetus that she was carrying at the time of the interview. Rather considerations of an abortion established an important link to her firstborn. The mere potentiality of an abortion challenged the relationship with this first child who had down syndrome and, thus the notion of unconditional love towards him.” (Reinsch et al.,* [22]*, p. 209).*

*“I think, for me, in particular, my husband has multiple sclerosis and I’ve got two children already and I’m a bit older and so it was very important for me… I was quite clear about the fact that I couldn’t take on caring for something knowing I might have to care for my husband in the future.” (study participant, Lewis et al.,* [20]*, p. 877).*

Several researchers found the parents they spoke with profoundly mourned the loss of meaningful social experiences or personal feelings they had an expectation they would experience during pregnancy or as parents before they received a T13/18 diagnosis.

*“These feelings contrast poignantly with a normal pregnancy experience, where social acknowledgment of the expected baby, even from strangers, adds to the joy of the pregnancy. The deep divide between the parents’ personal experience and the social experience was a source of pain beyond their feelings of grief and loss.” (Cote-Arsenault & Denney-Koelsch.,* [14]*, p. 1307).*

*“The ultrasound technician had a poker face like you would not believe. I had no clue that anything was wrong. I think that I would have preferred for her to have found a doctor and talked to me right there because we learned the sex of our baby and I had practically redecorated the nursery the night I found out, and we had called the whole family.” (study participant, Walker* et al.*, *[24]*, p. 15).*

#### Significance of time

The studies reviewed discuss time in diverse and meaningful ways. In this theme, we express our understanding that time was discussed as both indicative of states of being and a thing to be hoped for and cherished. The first way we understood time to be meaningful to the moral experiences of families within the context of T13/18 was the way in which many researchers, explicitly through participant testimony, described the isolation and disempowerment parents felt navigating the medical system.

*“We were in limbo.” (study participant, Cote-Arsenault & Denney-Koelsch.,* [14]*, p. 1305).*

*“Parents heard bad news over time, in disconnected, uncomfortable interactions from different providers. Diagnoses often took many weeks, leaving the mother and father anxious and worried, but still hoping for the best.” (Cote-Arsenault & Denney-Koelsch.,* [14]*, p. 1306).*

Findings and participant testimony presented by researchers also reflected discontinuity in care was a significant factor leading families to experience distress, confusion, frustration, and uncertainty over the future choices they would have to make on behalf of their infant.

*“Discontinuity of care was a common complaint among our study population. Families were often referred to a large medical centre or a high-risk obstetric clinic for further care.” (Walker et al.,* [24]*, p. 14).*

*“I lost contact with my regular health care provider, so we got shuttled into a little subsystem. It’s like ‘something’s wrong, so you don’t go to us anymore, you go to these new people.’ It would’ve been nice if my doctor would’ve called and said ‘you know I understand that there’s a problem, let me know if there’s anything I can help you with’.” (study participant, Walker et al.,* [24]*, p.14).*

Clinical spaces were not the only setting where researchers reported parents experienced feelings of powerlessness. Some parents also had a similar experience in online support groups.

*““A mother who chose hospice for her child described online resources as often being characterized by judgment from parents with surviving children towards those who didn’t choose aggressive interventions (followed by an excerpt from interview transcript)– “What you are going to see is all the parents that still have living trisomy kids are the ones that are out there saying, do this, do this, do this. It’s the rest of us, the 90%... we basically keep our mouth shut about it because that’s not what our story was.”” (Arthur and Gupta,* [3]*, p. 233).*

*“I looked on the Internet and found some support groups. They get people posting who have a spiritual view or whose babies live a long time. There is the tendency to think that that is the typical experience, which is a shortcoming of leaving the patient to find information on their own.” (study participant, Walker et al., *[24]*, p. 16).*

Parents were also highly future-oriented, some studies reported, keenly invested in thinking about how their choices in pregnancy would impact their ability to fulfill the interests of themselves, and their family in the future.

*“I believe continuing my pregnancy was beneficial to my long-term emotional health because it allowed a more natural grieving process (vs. termination).” (study participant, Guon et al.,* [5]*, p. 311).*

*“Participants also reflected on their ‘future lives’, for example, one participant with a high-risk reflected on what the long-term future of parenting a child affected by down syndrome [the study looked at a variety of trisomies] would look like and the implications for her own children” (Lewis et al.,* [20]*, p. 876).*

Time was not simply described by studies in terms of a state of being, it was also something parents hoped for deeply– time with their child.

*“My biggest hope was that he would not suffer in any way. Naturally I hoped that he would be with us for a long time and have a good life. I wanted to give him a chance.” (study participant, Janvier et al.,* [15]*, p. 281).*

Researchers across studies found a hope for time was often accompanied by some common desires of parents including: to be together as a family, to have their infant be born alive, and to bring them to their home.

*“I hoped that we would be able to bring her home and raise her and care for her in our family.” (study participant, Janvier* et al., [15], *p. 281).*

*“I would like to be able to bring her home and have a family experience having her home. I am hoping to let her have quality time with us as a family once we are able to get her to that point. I hope one day she will be well enough to go home with us to be a family for as long as she is able.” (study participant, Weaver et al.,* [27]*, p. 2).*

In cases where time with the fetus or newborn was likely to be critically short, multiple studies found parents were comforted by healthcare professionals who encouraged parents to reconsider the time they deemed as part of their child’s life.

*“They suggested that we treat Tessa’s time in the womb as part of her life. It made her life more meaningful to our younger children and us.” (study participant, Janvier et al.,* [17]*, p. 266).*

Some studies suggested parents experienced significant distress in cases where healthcare professionals did not encourage this outlook on life, time, and togetherness. This quote stood out as quite a visceral emotional description of a mother feeling able to bond with her child, and craft memories, only after the child had died, and no one cared what she did.

*“One mother struggled to recall a fond memory of the 14 weeks she spent with her child in the ‘sterile’ NICU because, “I didn’t feel like she was mine […] all of my pictures I’m in a hospital gown and no wedding ring… that’s not me.” After her daughter passed away, she finally had a chance to craft the memories that she wanted, “I wanted my picture taken because she wasn’t attached to anything […] and then I wouldn’t put her down for some hours […] because she was finally mine […] because she wasn’t connected and nobody cared what I did.” (study participant, Arthur and Gupta,* [3]*, p. 232).*

## Discussion

The persistence of the ethically ambiguous state of T13/18 in medical, social, and cultural communities, as demonstrated by our analysis of the literature we reviewed, has a profound influence on many families’ interpretation and sense of how their values may be both thwarted and actualized in their lived experiences of T13/18. In this discussion, we present a constructive reflection on how our inquiry into the moral experience of families captured in the current body of literature on their lived experiences contributes to a strengthened understanding of how we might view and navigate the invasiveness of these conditional states of persistent ethical ambiguity.

An inquiry into the agency of a particular group, as our theoretical lens understands it, involves the discernment of phenomena in terms of their broader meaningfulness [30]. From a moral experience lens, this process involves seeking to understand how the experience of this phenomenon (which may be conceived of events, interactions, interpretations, situations, circumstances) matters to the agent, which is in part shaped by the systems of meaning the agent is socially embedded within [8]. Our review collected a small but rich body of literature which explored the lived experiences of families in the context of T13/18. Our analysis demonstrated how (a) pride as resistance, (b) negotiating normalcy, and (c) the significance of time can be regarded as meaningful concepts to further our understanding of how agency is perceived and exercised by families experiencing T13/18 as expressed by the body of literature we reviewed.

Our theme, ‘pride as resistance,’ acknowledged the myriad of different ways that experiences and normative evaluation interrelate in the context of T13/18 and suggests some families who chose to continue their pregnancy found deep meaning in resisting the historically dominant discourse in medicine that T13/18 are conditions incompatible with life. In our analysis, we discussed the agentic nature of this resistance, reflected in the fighting metaphors both authors and their participants used [3]. Resistance, especially of a nature that families in the literature expressed, presupposes the existence of a strong ‘force’ one interprets as working against them. This lens allows us to unpack why families who wish to continue their pregnancy might feel the need to be zealous in their advocacy publicly and in online support forums for other parents. One could also argue studies that pointed out the use of fighting metaphors are reflective of families’ cultural beliefs about engaging in parenthood. This resistance may support the ability of parents to express how meaningful it is for their child’s agency, personhood, unique attributes, and life story to be recognized by individuals and communities they are or seek to be embedded within. Equally, taking on that advocacy role can be exhausting, isolating, and increase perceptions of injustice. There is a distinct richness to the moral nature of the experiences captured in the literature we reviewed, which describes families experiencing many emotions and expressions of identity like pride, grief, surprise, play, anger, resistance, sass, acceptance, spunk, magnetism, peace, and toughness, in complex and intertwined ways.

Our theme, ‘negotiating normalcy,’ discussed families’ experiences of desiring and, in some cases, sensing they were denied access to culturally valuable ‘goods’ within their social communities— expressed in terms of beliefs and values— like the loss of a ‘normal’ pregnancy experience. Families in the studies we reviewed experienced the ethical ambiguity associated with T13/18 as invasive in their social interactions [3, 15, 24]. This invasiveness challenged their access to social acknowledgments that mattered to their sense of social belonging and to the formation of their identity as parents ‘in becoming’. We also discuss critical ideas in the literature we reviewed about the underappreciation of how morally and existentially challenging prenatal screening can be for families. Parents after a T13/18 diagnosis may be vulnerable to pressures regarding what identity they must take up to be a ‘good parent’ considering the new knowledge they have acquired through the diagnosis. In the current available research this is mainly explored through studies that looked at parents’ involvement in social networks [16], or studies that discuss how parents navigated the interpretation of trustworthy informational sources [24]. As research about ‘good parent beliefs’ evolves and grows in the pediatric ethics space [31, 32] this could be a powerful avenue into future research on the moral experiences of families in the T13/18 context. In the context of what we know about persistent ethical states of ambiguity, we might consider the lack of acknowledgment of how challenging the screening process can be for families, which could contribute to the moral distress they face in the decision-making process that follows their screening.

Our theme, the ‘significance of time,’ analyzed how time, both temporally and spatially, was morally significant to how parents/families conceptualized their agency and ability to make normative evaluations about decisions on behalf of their pregnancy/child. Looking at both the temporal and spatial expression of time offered unique ways to think about how parents assess information and express their hopes and desires for their child in the context of T13/18. Several philosophical scholarly contributions on the ethical challenges surrounding the provision of life-sustaining measures in cases of T13/18 motivate a central tension in these cases concerns how a family and clinical team conceive of choices in the best interest of an infant considering their predicted present and future quality of life. In healthcare, quality and quantity of life can often be positioned as dichotomous concepts in goals of care discussions [33]. Across multiple studies included in this review [5, 17, 27], families expressed the prominence of this dichotomy (that their choices in effect would either attend to quality or quantity of life concerns, not both at the same time) in medical cultures constrained their ability to feel heard and understood by clinicians in establishing goals of care for their child. Families often felt their motivations to push for life-sustaining treatment were mischaracterized by clinicians as efforts to simply prolong their child’s life or a failure to appreciate the severity of their infant’s condition.

Several studies we reviewed [15, 18] provided rich insight that a crucial part of families’ moral experience was a deep desire for engagement and connection with their infant, to make memories and bonds outside of clinical environments many parents felt alienated within. This might necessitate more attention in the clinical setting be given to supporting a family to consider how they integrate their infant with T13/18 into the quality of being a member of their family in a way that feels real and meaningful considering their unique moral, cultural and/or spiritual context. The current body of research positions quality of life, quantity of life, and the value of ‘togetherness’ or the ‘adoption’ of the child into the existing family of parents as significant values that are often held by parents simultaneously. In exploring the significance of these values to the moral experience of families, one might consider how a parent’s motivation to pursue ‘togetherness’ as an interest could influence prioritization of this value ahead of other values perceived to be more constitutive to the best interest of the child. However, the social and cultural complexities of familial interests demonstrated within this body of literature illustrate separating the interests of a critically ill infant from those of their parents is ethically and practically challenging in a condition like T13/18. This insight is also reflective of growing ethical discourse [34–36] in associated bodies of literature on the utility of the best interest of the child standard in establishing goals of care for children across pediatric medical specialties.

Of critical importance to this discussion is the recognition that societal, cultural, religious, political, and legal factors influence the way different families consider the morality of their healthcare decisions and sometimes the authority and choices they can make on behalf of their child. In the context of this literature review, as the majority of articles included reported on studies conducted in America, a North American context bears relevance to the experiences of those research participants, the authors who reported on them, and our secondary analysis of that reporting. One study included in our review, which was conducted in Japan [18], points out in their clinical experience it appeared more natural for Japanese healthcare professionals in treating children with severe disabilities to consider their existence to be precious and valuable. Moral matters are rooted in a person’s moral ontology, that is, the underlying commonly implicit beliefs held by a person or a group of persons. The moral experience lens we adopted in this review recognizes these forces as ‘horizons of significance’ [8]. If we consider how families as moral agents stand against a background of ‘horizons of significance,’ we can start to distinguish how their agency is constructed by the context that they reside within and the sociohistorical-based moral order within which the meaning of their identity and experiences of having a pregnancy/child with a diagnosis of T13/18 is rooted [8]. The literature we analyzed in this review demonstrates having a pregnancy/child with a T13/18 diagnosis requires families to be in a complex process of continual appraisal of how their concerns stand in relation to their local moral context; this moral experience is compounded by the ethically ambiguous nature of their local moral context in relation to the conditions.

Empirical bioethical inquiry centring on how an individual or group exercises, or ‘ought’ to exercise, agency, can often presuppose a normative ethical lens seeking to understand how ethical principles or standards stand in relation to culturally accepted moral values we deem relevant to decision-making [8]. In this way, these lines of inquiry value the perceived benefit of exploring subjective and collective endorsements of moral systems constructed of coherent, systematic, and reasonable principles, rules, ideals, and values to produce ethically justified decision-making [8]. We would suggest conditions that find themselves in persistent states of ethical ambiguity (like T13/18) are uniquely suited to challenge the value of this presupposition. As such, researchers should consider the utility of alternative approaches to studying the contours of agency and, consequently, the nature of decision-making in these states.

In our review, we used a moral experience lens to analyze literature that explored the lived experience of families navigating a condition (T13/18) in a state of persistent ethical ambiguity. This approach allowed us to shift our focus from considering normative questions like, *“what does the literature say we should do to support parents to make ethical decisions in the context of T13/18?”* to *“what does the literature say about how parents’ interpretation of their experiences in the context of T13/18 inform what matters or is at stake for them in their decisions?”* and *“how are parents’/families’ experience of evaluating morality in their decisions shaped by the systems of meaning they are embedded in?– furthermore, do families have the power to resist or transform those systems of meaning?”* Normative questions ask what we should or ought to do. In states of persistent ethical ambiguity, while, of course, actions still need to be taken, just by the nature of these states themselves, we can presuppose that the “should” is highly resistant to an objective answer. We found in our review of the literature that a focus on moral experience and the systems of meaning that influence those subjective experiences was a rich lens to contribute applied and theoretical insight into why T13/18 remains in a state of persistent ethical ambiguity, and how families live and make decisions within that state.

### Strengths and limitations

It should be acknowledged this review only considered the inclusion of articles written or available in the English language. Regional legal regulations, cultural values, and healthcare policies in different regions bear significant context to our analysis. As the majority of articles in this review were conducted in the United States, it should be recognized our analysis may be more relevant to healthcare systems and bioethics traditions that bear similarities to a North American context. This review excluded normative literature on the subject, despite its valuable contributions to the ethical discourses we explore in our review. We made this decision to forefront empirical work which examined the experiences of families in the context of trisomy 13/18. Our review and analysis does not contend to speak to, or for, the moral experiences of all families in the context of T13/18. Consistent with our theoretical outlook our inquiry acknowledges this would be an impossible feat considering the subjective and culturally embedded nature of moral experience.

## Conclusion

This review suggests there is great value in applying a moral experience lens to research the experiences of families’ in the context of T13/18. Decision-making in the context of T13/18 engages with questions of the morality of prolonging life, suffering and the effects of illness and disability on a family. Our review demonstrates a myriad of other ethically relevant concerns deserve consideration in these discussions, including notions of a ‘normal’ pregnancy and child, the psychological price of continued resistance against a medical system one perceives themselves to be at odds with, how pride and resistance interrelate, how the decision-makers in families forecast their future needs, and how both quality and quantity of life can be complementary desires, among several others. The richness of these ethically relevant concerns demonstrates more research into the experience of families’ in the context of T13/18 could produce valuable insight into supportive actions to alleviate some of the moral distress these families, and their healthcare providers face.

## Data Availability

The articles reviewed in this scoping review are available in the associated journals in which they were originally published. Full citation details of these articles are included in the reference section.

## Abbreviations

T13/18: Trisomy 13 and/or 18
T13: Trisomy 13
T18: Trisomy 18

## Footnotes

1 I﻿n the case of T13/18 the patient group can include the individual carrying the pregnancy with a diagnosis of T13/18 and the infant born with T13/18. This paper reviews several articles, wherein many parents also recognized the fetus involved in the pregnancy as their child. In this paper we do not engage in philosophical, or political discussion, on the personhood status of a fetus. The articles we review in this paper also do not explore these nuances.

2 We choose the term family (or families) to refer comprehensively to a group of individuals which may (or may not include) the individual carrying a pregnancy with a T13/18 diagnosis, their partner or support system, the parents associated with the pregnancy/child, the pregnancy itself, or the child/infant itself after birth. Since we review studies which involve individuals who are navigating the context of T13/18 in different phases (pregnancy, after birth or after the death of their child), and therefore might constitute the members of their families in different ways, there is no clear linguistic convention to refer to all these individuals and states.
